# Development of a ribonuclease containing a G4-specific binding motif for programmable RNA cleavage

**DOI:** 10.1038/s41598-019-42143-8

**Published:** 2019-05-15

**Authors:** Dung Thanh Dang, Anh Tuân Phan

**Affiliations:** 1 School of Physical and Mathematical Sciences, Nanyang Technological University, Singapore, Singapore; 2 Faculty of Biotechnology, Ho Chi Minh City Open University, Ho Chi Minh City, Vietnam

**Keywords:** Catalytic RNA, Protein design

## Abstract

We developed a ribonuclease for site-specific targeting and cleavage of single-stranded RNA. The engineered RNase protein was constructed by incorporating two independent functional domains, an RNase HI domain that could cleave the RNA strand in a DNA-RNA hybrid, and a domain of the RHAU protein that could selectively recognize a parallel DNA G-quadruplex (G4). The newly designed RNase first recruits a DNA guide oligonucleotide containing both a parallel G4 motif and a template sequence complementary to the target RNA. This RNase:DNA complex targets and efficiently cleaves the single-stranded RNA in a site-specific manner. A major cleavage site occurs at the RNA region that is complementary to the DNA template sequence. The newly designed RNase can serve as a simple tool for RNA manipulation and probing RNA structure.

## Introduction

RNA plays a central role in various biological processes including gene expression, gene regulation and catalysis^1–4^. Many RNA transcripts are of several thousand nucleotides long and understanding the structure and function of full-length RNA is not always possible. Thus, fragmentation of the RNA molecules is an attractive approach for RNA research^5^. RNA-based enzymes (ribozymes), which can catalyse RNA cleavage in a sequence-specific manner, include artificially selected ribozymes and natural ribozymes, such as those involved in RNA splicing and RNA processing^6,7^. In addition, development of DNAzymes for RNA cleavage has also been reported^8^. However, the use of protein enzymes (ribonucleases or RNases) for fragmentation of the RNA molecules is a wide-spread method^5^. Unlike Type II restriction enzymes that cleave DNA in a site-specific fashion^9^, most of RNases exhibit non-specific cleavage activity^5,10^.

Development of RNase systems for site-specific RNA cleavage would greatly help the studies of RNA structure and function. An artificial site-specific RNA endonuclease was generated by incorporating an RNA cleavage domain with a series of Pumilio/fem-3-binding factor domains that could specifically recognize an 8-nt RNA sequence and cleave near the binding site^11^. RNase H enzymes, consisting of a RNA-DNA hybrid binding domain and a catalytic domain, can cleave the RNA strand in a RNA-DNA hybrid^12^. Fusing the catalytic domain RNase HI with a sequence-specific zinc-finger protein resulted in an enzyme that cleaved the RNA strand in a RNA-DNA hybrid at 5 nt from the recognition sequence^5^. However, this technique will require the specific nucleotide sequence to be present at the desired location of the RNA molecule. DNA oligonucleotide-targeted RNA cleavage provides an alternative approach for fragmentation of RNA molecules^13–19^. Hybridization of a short DNA oligonucleotide was early proposed to direct the RNase H-mediated cleavage at a specific site of a RNA molecule^13^. Later, RNase H was used for site-specific RNA cleavage using the complementary chimeric 2′-*O*-methyl^15,20^ DNA or 2′-*O*-methyl RNA-flanked DNA^21^. To enhance the RNA site-specific cleavage efficiency, a short antisense DNA oligonucleotide was covalently conjugated to RNase H to generate a hybrid ribonuclease^18,19^. Especially, the RNase H-antisense oligonucleotide conjugate was shown to efficiently cleave target RNA transcripts and inhibit gene expression *in vitro* and in living cells^18,19^. In this approach, the RNase H-antisense oligonucleotide chemical conjugation is the limiting step for varying target sites^5^.

It has been established that RNA can act as a template for a ribonucleoprotein to selectively recognize and knockout complementary messenger RNA (mRNA) including the miRNA and siRNA machineries^22,23^. Recently, powerful CRISPR-Cas systems have been applied for RNA recognition and cleavage. The crisprRNA-Cas protein complex can directly recognize and cleave target RNAs^24^. The type VI effector Cas13 acts as a crRNA-guided ribonuclease function which can degrade RNAs^25^. The type III-associated RNase (Csm3 and Csm6) targets and cleaves the viral DNA and its transcriptional RNAs^26^. Programmable RNA recognition and site-specific cleavage by Cas9 has been reported^27,28^. By using specifically designed DNA PAMmers, the Cas9 system can selectively bind and cleave RNA targets in a site-specific manner *in vitro*^27^. Without addition of DNA PAMers, recognition of a natural PAMer of RNA target by NmeCas9 also allows site-specific cleavage of the RNA target^28^. Despite these advances, it is not simple to manipulate these CRISPR-Cas systems for programmable RNA recognition and cleavage due to the protein complexity and a need for guide RNA, additive DNA-PAMer or rPAM presence. This study aimed to establish a simple ribonuclease approach for programmable site-specific RNA cleavage, based on a DNA guide and protein-DNA structure-specific recognition.

G-quadruplexes (G4) are four-stranded structures formed by stacking of multiple G-tetrads^29,30^. G4 structures are highly polymorphic: four strands of the G-tetrad core can be parallel (oriented in the same direction) or non-parallel^31,32^. In cell, the formation of G4 structures has been implicated in many biological processes such as telomere maintenance, replication, transcription and translation^33,34^. G4 structure provides a potential target site for recognition by small molecules and proteins^35,36^. The specific interaction between the N-terminal domain of the RHAU protein and a G4 has been characterized^37,38^. Incorporating fluorescent protein to this RHAU peptide motif provided a useful probe for discrimination between different G4 topologies^39^.

In this study we generate a novel RNase, when formed a complex with a guide DNA strand containing both a parallel G4 motif and a template sequence, is capable of site-specific cleavage of a single-stranded RNA at a predetermined position, providing a powerful tool for RNA manipulation.

## Results

**Figure 1:**
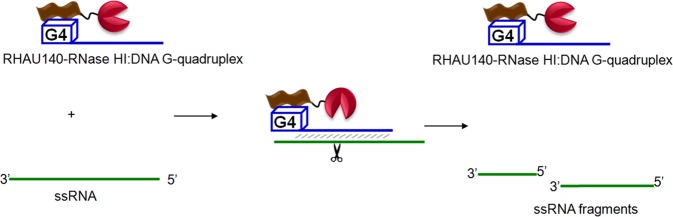
Construction of a G4-specific RNase for programmable site-specific RNA cleavage. The newly designed ribonuclease is composed of the RHAU140 domain (coloured in brown) and the RNase HI domain (coloured in red). RHAU140-RNase HI forms a complex with a short DNA guide bearing a parallel G4 motif (blue). This complex selectively targets a single strand RNA (green) and then site-specifically cleaves the RNA strand at the DNA-RNA hybridization site.

It was shown that while a short 16-aa RHAU recognition motif (aa 53–68) is sufficient for specific recognition of a parallel G4, longer RHAU peptides exhibit higher binding affinity with RHAU140 (aa 53–192) displaying a dissociation constant (*K*_*d*_) in the nanomolar range^37,39^ possibly due to the packing of several helices formed at the N-terminal of RHAU^40^. We designed a ribonuclease RHAU140-RNase HI by incorporating a catalytic domain of RNase HI from *B*. *halodurans* to the RHAU140 peptide. The newly designed RNase is expected to be capable of recruiting a DNA strand containing both a parallel G4 motif and an antisense DNA template sequence. The RNase:DNA complex can selectively target and cleave a single RNA strand at the predetermined position that is complimentary to the template DNA (Fig. 1).

Two RNase proteins, the hybrid RHAU140-RNase HI and the control wild-type RNase HI catalytic domain (Supplementary Table S1), were cloned, expressed and purified. RNases with good purity was obtained as monitored by SDS-PAGE (Supplementary Fig. S2).

To determine whether the fusion protein RHAU140-RNase HI and the DNA strand bearing a parallel G4 motif formed a complex, a binding assay was performed by gel electrophoresis mobility shift assay (Supplementary Fig. S3). RHAU140-RNase HI was observed to bind the DNA strand B1 containing a parallel G4 motif (Table 1) causing a change in the B1 mobility in the gel. In a negative control, upon addition of the same amount of RHAU140-RNase HI to B0 (a DNA strand without a G4 motif) (Table 1), no change in the mobility of B0 was observed. The wild-type RNase HI did not show any binding to B1 and B0 (Supplementary Fig. S3). Note that the RNase HI construct used contains only the catalytic domain and lacks the nucleic acid binding N-terminal domain. The selective recognition of a parallel G4 by RHAU140 allowed RHAU140-RNase HI to form a complex with DNA bearing a parallel G4 motif, bringing the RNase HI domain close to the RNA substrate for enhancement of catalytic activity.

**Table 1:** Oligonucleotide sequences used in this study.

Name	Sequence
R1	5′-FAM-CCAUGAUGAUGCCGUCGUCGUUGAAGUCUU-3′
B1	5′-TTGGGTGGGTGGGTGGGTTCAACGACGACGGCATCATC-3′
B0	5′-TTGACTAAGTGCCTTAGTTCAACGACGACGGCATCATC-3′
C1	5′-TTGGGTGGGTGGGTGGGTCGACGACGGCATCATCATGG-3′
D1	5′-TTGGGTGGGTGGGTGGGTAAGACTTCAACGACGACGGC-3′
E1	5′-TTGGGTGGGTGGGTGGGT**TTTT**TCAACGACGACGGCATCATC-3′
F1	5′*-*TGGGTGGGTGGGTGGGT**TTTTTTTT**TCAACGACGACGGCATCAT-3′

R1 is RNA while other sequences are DNA. The G4-forming motif is underlined. T_4_ and T_8_ linkers between the G4 motif and the template sequence are shown in boldface.

**Figure 2:**
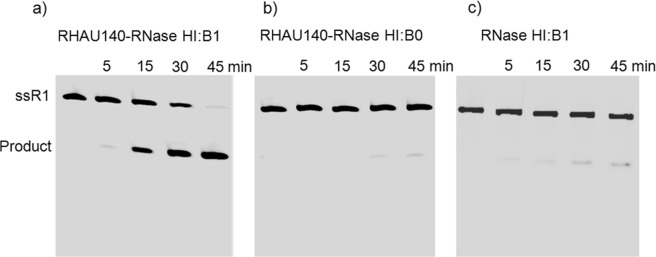
Cleavage of the RNA strand R1 by using the complexes of (**a**) RHAU140-RNase HI:B1, (**b**) RHAU140-RNase HI:B0 and (**c**) RNase HI:B1. The RNA strand R1 was labelled with FAM at the 5′-end. Reactions were carried out by incubation of RHAU140-RNase HI:B1 (0.3 µM:0.3 µM), RHAU140-RNase HI:B0 (0.3 µM:0.3 µM) and RNase HI:B1 (0.3 µM:0.3 µM) with R1 (10 µM) in reaction buffer at 37 °C. The samples which were collected after 5, 15, 30 and 45 minutes were analysed by using a denaturing gel with 7 M urea.

The RNA cleavage activity of RNase:DNA complexes was tested. The RNA substrate R1 was labelled with Fluorescein (FAM) at the 5′ end (Table 1). The complexes of the RNase and DNA oligonucleotides were well mixed at the 1:1 ratio before adding to the reaction. Incubation of the RHAU140-RNase HI:B1 complex with R1 showed that the cleavage product of R1 was increased in a time-dependent manner (Supplementary Fig. S4). It took around 45 minutes for RHAU140-RNase HI:B1 (0.3 µM:0.3 µM) to fully cleave the substrate R1 (10 µM) (Fig. 2a). A specific cleavage site occurred inside of the complementary region between RNA and DNA (see below). Control experiments using the same amount of mixture of RHAU140-RNase HI:B0 (Fig. 2b) or RNase HI:B1 (Fig. 2c) showed only minor cleavage (at the same cleavage site) after the same incubation time with R1. These results revealed that RHAU140-RNase HI:B1 can specifically target and efficiently cleave R1. The complex of RHAU140-RNase HI:DNA consisting of a G4 motif would provide a powerful tool for direct cleavage of RNA at a predetermined position.

**Figure 3:**
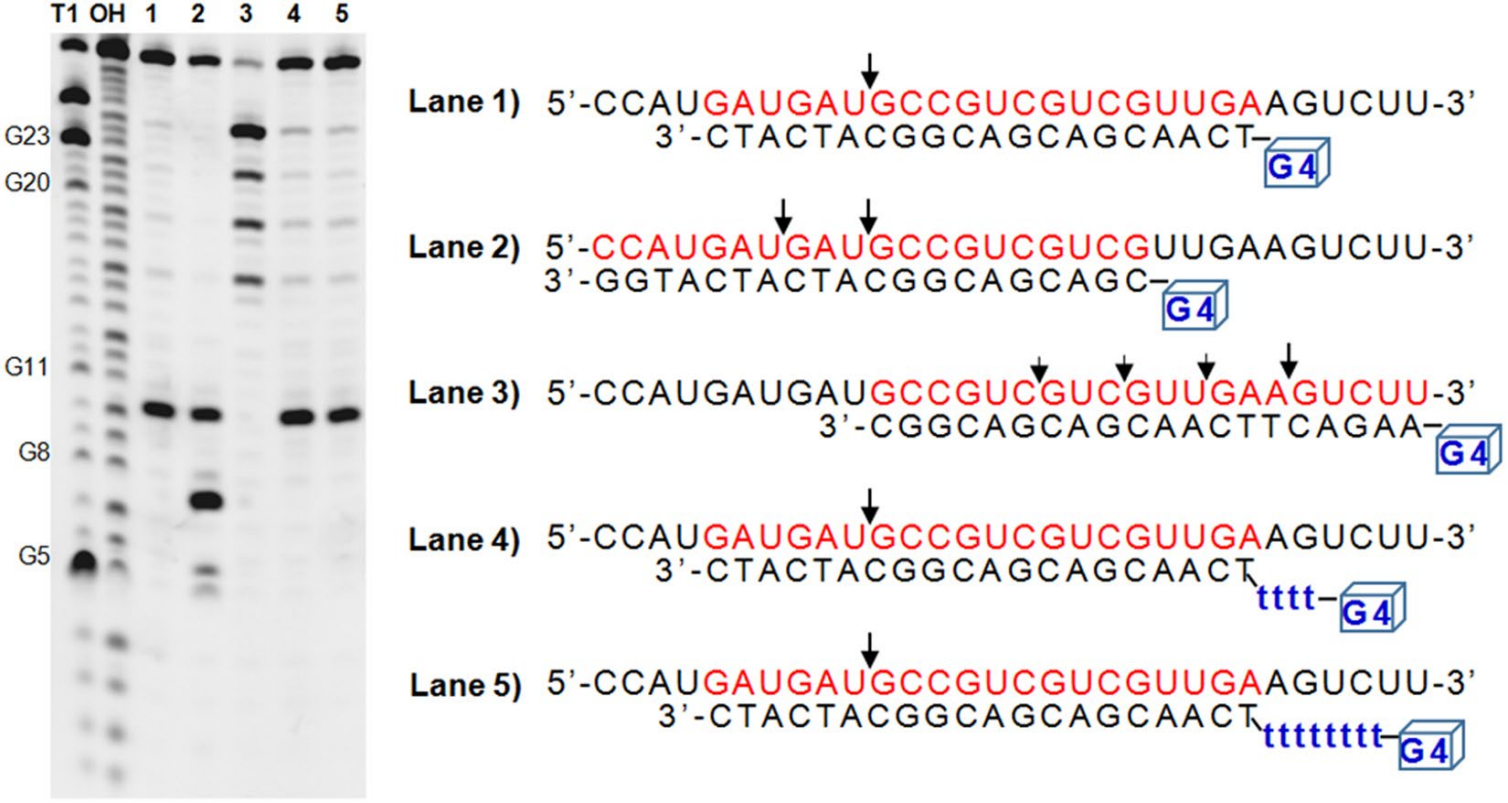
Cleavage site mapping on the RNA strand R1 by the complexes of RHAU140-RNase HI with variants of guide DNA oligonucleotides: B1, C1, D1, E1 and F1. Reactions were carried out by incubation of 10 µM R1 with complexes of 0.3 µM RHAU140-RNase HI and 0.3 µM each DNA oligonucleotide (B1, C1, D1, E1 and F1) in the reaction buffer at 37 °C. All of samples were subjected to a long denaturing gel with 7 M urea. “T1” indicates RNase T1 cleavage of R1 and “OH” indicates alkaline hydrolysis. Lanes 1, 2, 3, 4 and 5 are indicated by RNA cleavage by the complexes of RHAU140-RNase HI and different DNA oligonucleotides B1, C1, D1, E1 and F1, respectively. The main cleavage sites are indicated by arrows. The parallel G4 motif (sequence, 5′-TTGGGTGGGTGGGTGGGT-3′) is shown schematically as a small blue cube.

Cleavage site mapping of the complexes of RHAU140-RNase HI with different DNA oligonucleotides consisting of a G4 motif (B1, C1, D1, E1 and F1) have also been studied (Table 1). B1 was designed as a DNA oligonucleotide consisting of 2 parts: a complementary template sequence (20 nt) and a G4 motif without linker in between. E1 and F1 had an additional linker of 4 thymines (T_4_) and 8 thymines (T_8_), respectively, between the G4 motif and the template sequence (Table 1). C1 and D1 were designed to target different region of R1. Data in Fig. 3 showed that complexes of RHAU140-RNase HI:B1, RHAU140-RNase HI:E1 and RHAU140-RNase HI:F1 selectively targeted and specifically cleaved R1 mainly at the same cleavage position (6 nt away from the 5′ of RNA in the DNA-RNA hybrid) providing a major product (lane 1, lane 4 and lane 5, respectively). These results demonstrated that the linker between the G4 motif and the template sequence did not affect the cleavage site. The complexes of RHAU140-RNase HI:C1 and RHAU140-RNase HI:D1 cleaved R1 at several positions (RHAU140-RNase HI:C1 at 7 nt and 10 nt (lane 2); RHAU140-RNase HI:D1 at 6 nt, 9 nt, 12 nt and 15 nt (lane 3) away from 5′ of RNA in the DNA-RNA hybrid) providing several fragments. Results showed that the cleavage site depended on the nucleotide sequences of DNA-RNA hybrids. Interestingly, all of cleavage sites in our assays were at a 5′-guanine.

## Discussion

Development of a novel ribonuclease by fusing separate functional protein domains is a useful approach for programmable site-specific RNA recognition and cleavage. We generated a recombinant ribonuclease, RHAU140-RNase HI by using the catalytic domain of RNase HI and the G4-recognition domain RHAU140. The engineered RHAU140-RNase HI protein selectively binds to a short antisense guide DNA bearing a parallel G4 motif (G4-DNA), leading to the formation of a complex of RHAU140-RNase HI:G4-DNA. This complex can directly bind and cleave an RNA target at a predetermined position.

In fact, RNase H family itself is able to target and cleave the RNA strand of a DNA-RNA hybrid^12^. However, the antisense DNA oligonucleotide and RNase components need to co-localize at the desired cleavage site of the RNA molecule for the reaction to happen, hence, limiting the efficiency of site-specific RNA cleavage. Pre-loading of the DNA template on the enzyme, such as in a DNA oligonucleotide-conjugated RNase H, was shown to enhance the direct binding and site-specific cleavage of the RNA target^18,19^. However, this approach requires chemical conjugation between the DNA template and the RNase H enzyme. On the other hand, the systems of a zinc-finger protein conjugated RNase^5^ and an artificial RNA endonuclease^11^, while having the advantage of being protein-only sequence-specific RNases, would require particular RNA sequences at the desired locations of the RNA substrate. Sequence-specific ribonuclease systems using non-covalently bound RNA templates including miRNA, siRNA and crRNA are powerful and have been exploited both by the nature and in biotechnology^41^. Our approach is similar regarding the use of a nucleic acid template. However, while the specificity and efficiency might not meet that of the natural systems, its simplicity and the use of a more robust and inexpensive DNA template could make our approach attractive.

The complex of RHAU140-RNase HI:G4-DNA acts as a nucleoprotein enzyme to catalyze the site-specific RNA cleavage. Major cleavage sites occur inside the RNA region that is complementary to the DNA template and depend on nucleotide sequence of the DNA-RNA hybrid^42,43^. All of major cleavage sites in our assays were at a 5′-guanine, consistent with a previous report on multiple nucleotide preferences of the cleavage sites of RNase H^43^. The linker between RHAU140 and the RNase HI domain may also affect the position of the cleavage site(s)^5^. However, the effect of the linker between the G4 motif and the template region was not observed. The site-specific cleavage position can be further restrained and optimized by using a DNA guide strand containing modified nucleotides^20^.

Generally, RNase HI can cleave non-specifically any RNA sequence in a DNA-RNA hybrid. Thus, in principle any RNA segment that is unique in the RNA molecule can be chosen as the cleavage target in our approach. On the other hand, the cleavage efficiency and specific cleavage site(s) depend on the sequence of the chosen target segment. Based on previous studies^42,43^, a RNA target sequence with highly-efficient cleavage site(s) can be chosen for programmable RNA cleavage. The cleavage site can be further restricted with a chimeric design of the DNA template segment^15,20^. In our approach, the DNA guide can be easily constructed by adding a DNA template sequence, complementary to the RNA target segment, to a stable parallel G4 motif, e.g. TT(G_3_T)_4_, not complementary to any part of the RNA molecule. This G4-DNA guide, forming a complex with RHAU140-RNase HI, can direct an efficient cleavage of the single-stranded RNA target in a site-specific manner. The cleavage of the RNA strand can be readily monitored and demonstrated by gel electrophoresis.

This study takes advantage of a G4-recognition motif of RHAU to construct an RNase that can recognize a G4-containing DNA template for programmable site-specific RNA cleavage. We have demonstrated the concept using a G4-binding motif of RHAU with a 140-aa length and a nanomolar-range affinity (10 nM < *K*_*d*_ < 100 nM) to parallel G4s. In principle, a full-length catalytic dead mutant of RHAU with much higher affinity to G4s can also be used. However, the large size of the full-length RHAU (>1000 aa) might limit its applications. On the other hand, a short G4-recognition motif of RHAU can be further engineered to have a higher affinity to G4s. The newly developed RNase can also be used to probe the formation of G4 structures in RNA, when using DNA templates not containing a G4.

This approach can be extended to other G4-binding proteins^34^ or proteins recognizing other structural motifs such as Z-DNA^44^ and hairpin loop^45^, as well as small chemical motifs such as biotin^46^ and digoxigenin^47^.

## Conclusion

A novel RNase was developed to site-specifically target and cleave single-strand RNA, providing a powerful tool for RNA structure and function studies. The RNase was generated by incorporating the catalytic domain of RNase HI to the N-terminal domain of RHAU which can form a complex with a DNA guide strand bearing a parallel G4 motif and a template segment. The RNase:DNA complex can selectively target and efficiently cleave single-stranded RNA at the DNA-RNA hybrid position.

## Methods

### Construction, expression and purification of RHAU140-RNase HI and RNase HI

The Ribonuclease protein RHAU140-RNase HI was generated by incorporating the RHAU140 peptide to the RNase HI catalytic domain from *B*. *halodurans*. DNA encoding for RHAU140-RNase HI was synthesized by IDT Singapore Pte Ltd. It was amplified by PCR using a pair of primer ON1 (5′-gcgtggatccgtccatgcatcccgggcacctgaaag-3′) and ON2 (5′-accaactcgagctactttcgcccgtaatcggccttaatttcc-3′). The PCR product was then cloned into treated pET-Duet1 (Merck) at BamHI and XhoI, resulting in plasmid RHAU140-RNase HI. The wild-typeRNase HI was generated by PCR using a pair of primer ON3 (5′-agccaggatccgatgggcgcaaaagaggag-3′) and ON2 (5′-accaactcgagctactttcgcccgtaatcggccttaatttcc-3′). This PCR product was then cloned into treated vector pET-Duet1 (Merck) at BamHI and XhoI, resulting in plasmid RNase HI (Supplementary Fig. S1).

The plasmids of RHAU140-RNase HI and RNase HI were transformed into *E*.*coli* strain BL21 (DE3) for protein expression. The bacteria were cultured in LB medium containing 100 µg/ml of ampicillin and the cells were grown at 37 °C, shaking at 220 rpm to an OD_600_ of 0.6–0.8. Then, IPTG was added to a final concentration of 0.5 mM. The cells were incubated for 10 hours at 16 °C, shaking at 180 rpm before being harvested. The pellet was re-suspended into the BugBuster protein extraction reagent consisting of benzonase nuclease. The insoluble material was removed by centrifugation at 20,000 rpm, for 1 hour at 4 °C. The soluble fraction was applied to a column filled with Ni-agarose beads. The column was washed with 30 volumes of buffer (10 mM potassium phosphate, 100 mM potassium chloride, 10 mM imidazole, pH 7). The proteins were then eluted by buffer (10 mM potassium phosphate, 100 mM potassium chloride, 200 mM imidazole, pH 7) and the high concentration of imidazole was removed by dialysis buffer (10 mM potassium phosphate, 100 mM potassium chloride, pH 7). The purity of desired proteins was analysed by SDS-PAGE (Supplementary Fig. S2).

### Gel mobility shift assay

The gel mobility shift assay was performed using native PAGE of 10% polyacrylamide in 1X TBE (Tris-borate-EDTA), 20 mM potassium phosphate, 100 mM potassium chloride, pH 7.0. The DNA oligonucleotides, B1 containing a G4 motif (10 µM) and B0 without a G4 motif (10 µM) (Table 1), were incubated with 50 µM RHAU140-RNase HI or 50 µM RNase HI. The mixtures were subjected to the native PAGE and all the images were taken by using the Gel Doc UV shadowing technique (Alpha Innotech).

### RNA cleavage activity assay

The RNA target (R1), derived from the EGFP gene and consisting of 30 nt, was labelled with FAM at the 5′ end. DNA oligonucleotides B0, B1, C1, D1, E1 and F1 having 20 nt that are complementary to different parts of the RNA sequence. A G4-forming motif TT(G_3_T)_4_, which is not complementary to the RNA target, was introduced to B1, C1, D1, E1 and F1 (Table 1). All RNA and DNA oligonucleotides were purchased from IDT Singapore Pte Ltd. The complex of proteins and DNA oligonucleotides were well mixed at the 1:1 ratio before adding to the reaction. RNA cleavage activity assays were performed with 10 µM R1 in the 1X reaction buffer (50 mM Tris-HCL, 100 mM KCl, 3 mM MgCl_2_, 10 mM DTT, pH 8.3). A specific cleavage site occurred inside of the complementary region between RNA and DNA. Reaction was stopped by adding equal volume of formamide. For cleavage site mapping, T1 RNase and alkaline hydrolysis (Fermentas) cleavage was performed as RNA ladders. The samples were resolved on a 15% polyacrylamide-7 M urea gel and imaged by the Gel Doc (Alpha Innotech).

## Supplementary information

Supporting Information
