# Supplementary material for: Development of a ribonuclease containing a G4-specific binding motif for programmable RNA cleavage
Source: Sci Rep. 2019 May 15;9:7432. doi: 10.1038/s41598-019-42143-8 (PMC6520340; doi:10.1038/s41598-019-42143-8)
Supplement: Supplementary file 1 — Supporting Information [file 41598_2019_42143_MOESM1_ESM.pdf]

Development of a ribonuclease containing a G4-specific binding motif for programmable RNA cleavage

Dung Thanh Dang[1,2] and Anh Tuân Phan[1,*]

[1]School of Physical and Mathematical Sciences, Nanyang Technological University, Singapore

&

[2]Faculty of Biotechnology, Ho Chi Minh City Open University, Vietnam

Supporting Information

Supplementary Methods: Construction of RHAU140-RNase HI

The novel ribonuclease was genetically generated by incorporating human RHAU140 (aa 53-192) domain to RNase HI cleavage domain from B. *halodurans* with a flexible linker (SGGG)x4 in between. The pET Duet1 vector and PCR product of RHAU140-RNase HI (using a pair of primer: ON1/ON2) and RNase HI cleavage domain (using a pair of primer: ON3/ON2) were treated with *BamHI* and *XhoI* restriction enzymes. The treated RHAU140-RNase HI and RNase HI cleavage domain were then inserted into the treated vector by T4 ligase enzyme, resulting in the plasmid of RHAU140-RNase HI and RNase HI, respectively (Supporting Figure 1). All the genes were confirmed by DNA sequencing (data not shown). Amino acid sequences of RHAU140-RNase HI and RNase HI are shown in Supporting Table 1.

SDS gel experiments were performed using 12% gel acrylamide with 5% of stacking gel.

ON1: 5'- gcgtggatccgtccatgcatcccgggcacctgaaag -3'
ON2: 5'- accaactcgagctactttcgcccgtaatcggccttaatttcc -3'
ON3: 5'- agccaggatccgatgggcgcaaaagaggag-3'

Supporting Table S1. Sequences of RHAU140-RNase HI and RNase HI[a]

Proteins	Sequences
RHAU140-RNase HI	*mgsshhhhhhsqdpsm*hpghlkgreigmwyakkqgqknkeaerqeravvhmderreeqivqllnsvqakndkeseaqiswfapedhgygtevstkntpcsenkldiqekklinqekkmfrirnrsyidrdseyllqenepdgtldqklledlqkkk*sgggsgggsgggsgggqlv*gakeeiiweslsvdvgsqgnpgiveykgvdtktgevlferepipigtnnmgeflaivhglrylkernsrkpiysdsqtaikwvkdkkakstlvrneetaliwklvdeaeewlnthtyetpilkwqtdkwgeikadygrk
RNase HI	*mgsshhhhhhsqdpm*gakeeiiweslsvdvgsqgnpgiveykgvdtktgevlferepipigtnnmgeflaivhglrylkernsrkpiysdsqtaikwvkdkkakstlvrneetaliwklvdeaeewlnthtyetpilkwqtdkwgeikadygrk

[a]The His-tag sequences are shown in italic; the linker is shown in italic and underlined.

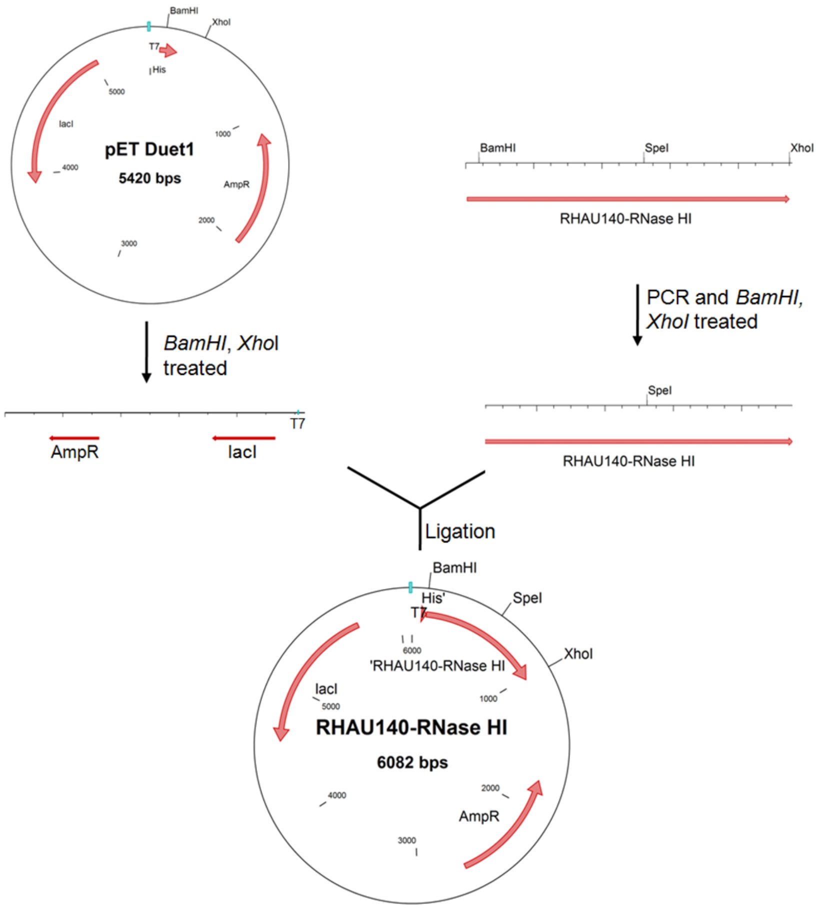

Supporting Figure S1. Construction of plasmid for expression of RHAU140-RNase HI.

(a)

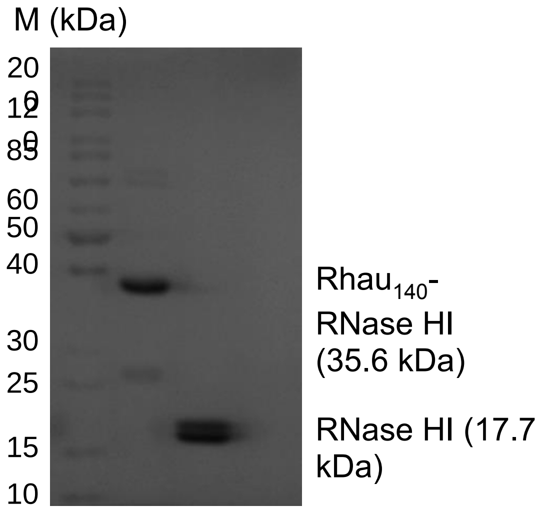

(b)

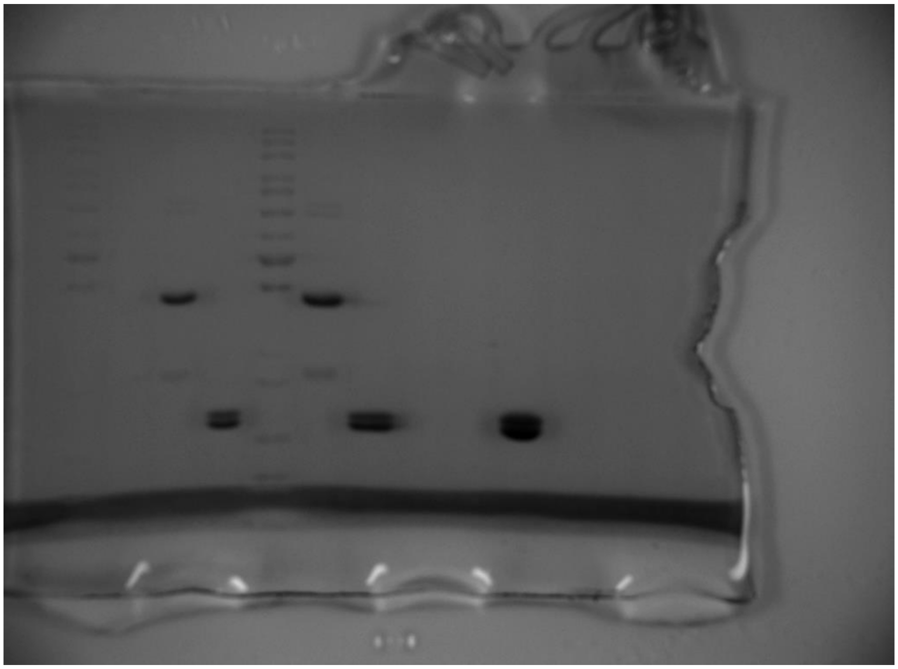

Supporting Figure S2. SDS gel of proteins. (a) and (b) are cropped and full picture, respectively.

(a)

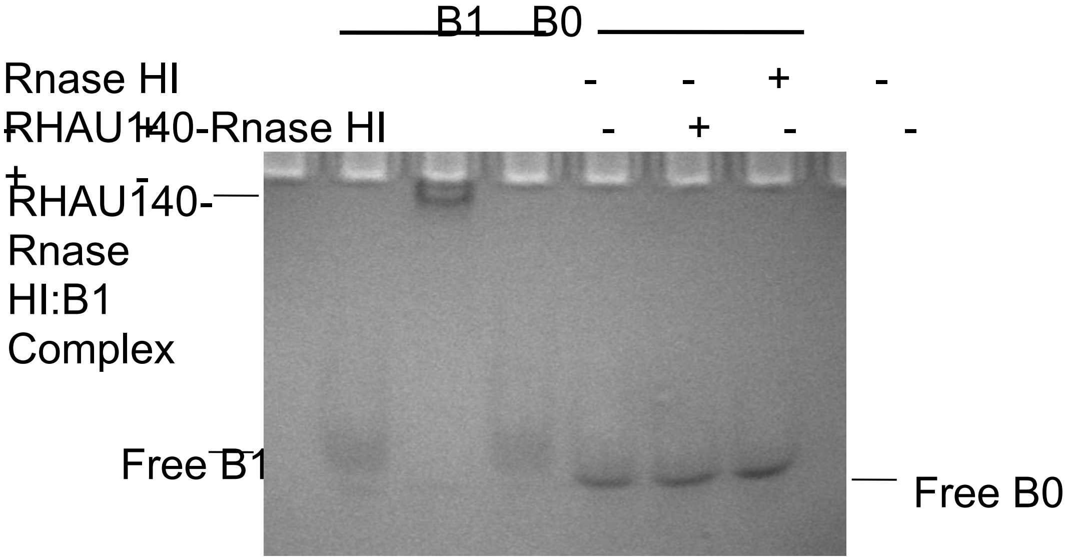

(b)

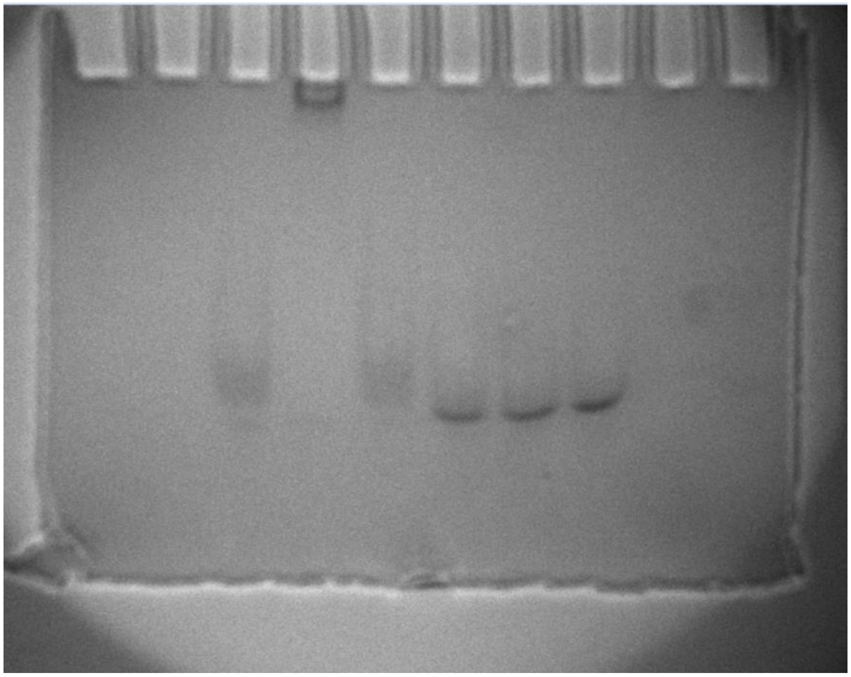

Supporting Figure S3. Gel mobility shift assay for binding of RHAU140-RNase HI and RNase HI with DNA oligonucleotides with a G4 motif (B1) and without a G4 motif (B0). Free 10 µM B1 or free 10 µM B0 and mixture with 50 µM RHAU140-RNase HI or 50 µM RNase HI were subjected to native PAGE. RHAU140-RNase HI fully bound B1 but not B0. RNase HI did not bind neither B1 nor B0. Images were taken by using UV shadow on the Gel Doc system (Alpha Innotech). (a) and (b) are cropped and full picture respectively.

a)

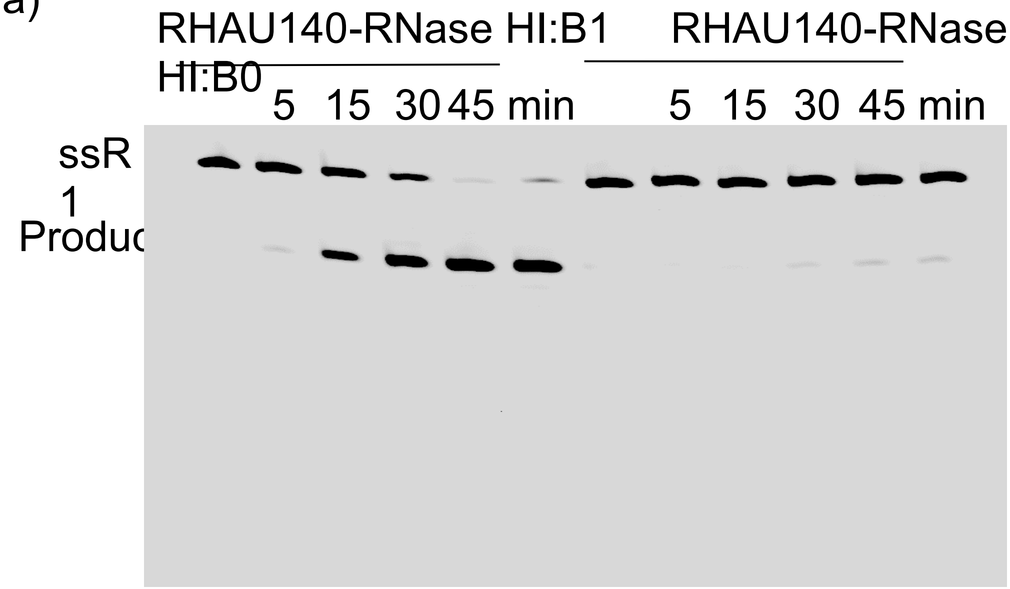
RHAU140-RNase HI:B1
RHAU140-RNase HI:B0
5 15 30 45 min
5 15 30 45 min
ssR 1
Produc

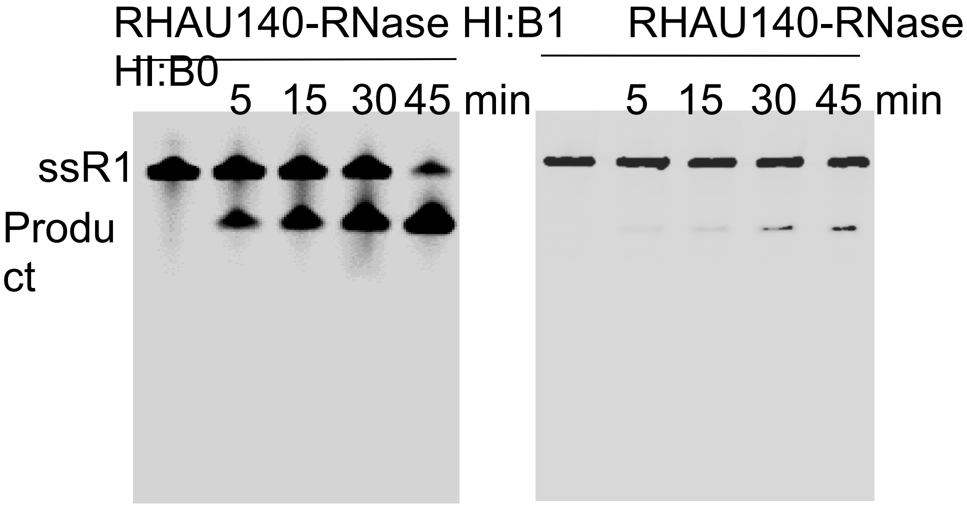
RHAU140-RNase HI:B1
RHAU140-RNase HI:B0
5 15 30 45 min
5 15 30 45 min
ssR1
Produ ct

b)

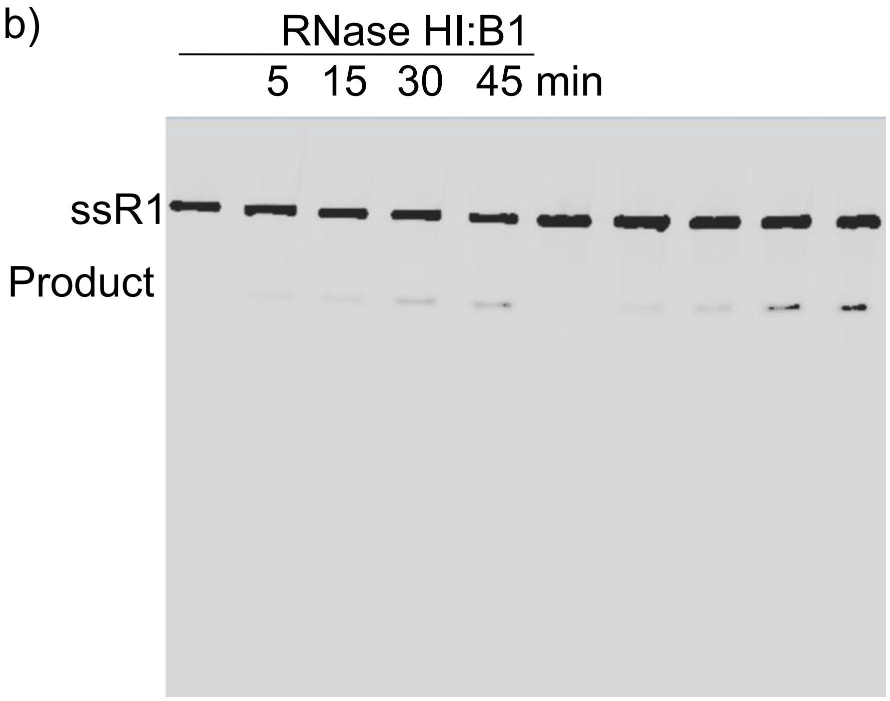

c)

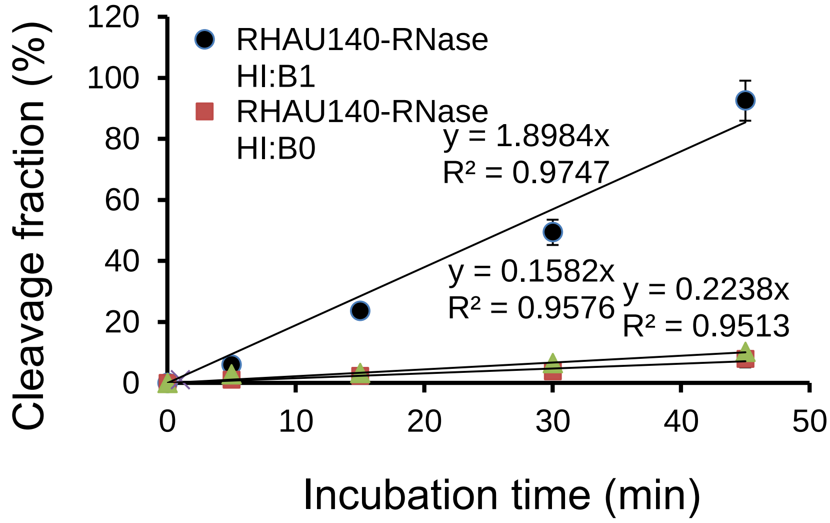

Supporting Figure S4. Uncropped gel picture of Figure 2. Cleavage of RNA strand R1 by using the complexes of (a) RHAU140-RNase HI:B1 (duplicate), RHAU140-RNase HI:B0 (duplicate) and (b) RNase HI:B1, (c) Time-dependent cleavage activity of RHAU140-RNase HI:B1 (circle), RHAU140-RNase HI:B0 (square) and RNase HI:B1 (triangle) were analyzed by the AlphaEaseFC software.

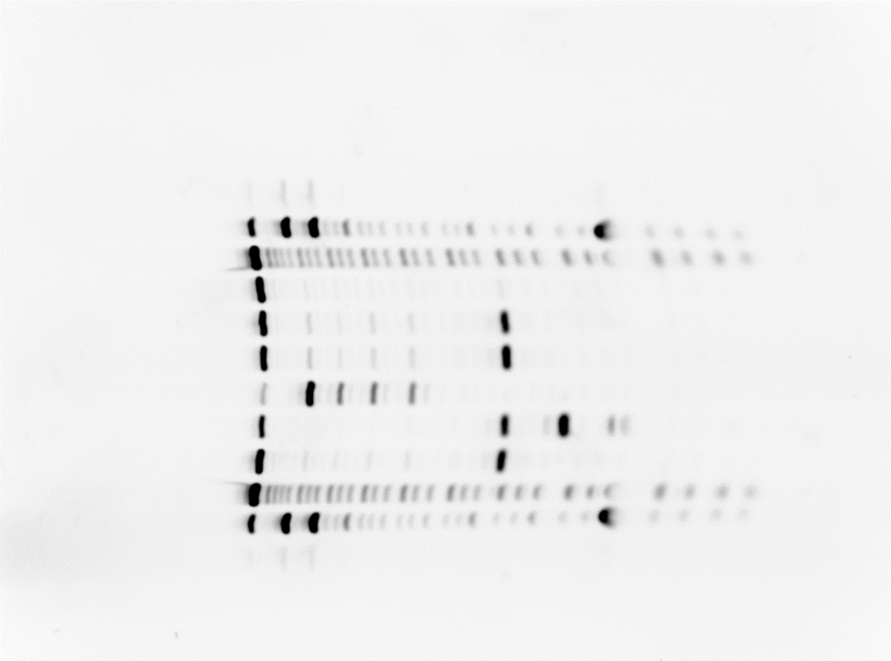

Supporting Figure S5. Uncropped gel picture of Figure 3. Cleavage site mapping on the RNA strand R1 by the complexes of RHAU140-RNase HI with variants of guide DNA oligonucleotides.
